# Capacity Estimation of Signalized Intersections Considering Connected Automated Vehicle Observability

**DOI:** 10.3390/s26020484

**Published:** 2026-01-11

**Authors:** Ruochuan Fan, Jian Lu

**Affiliations:** 1 Jiangsu Key Laboratory of Urban ITS, Southeast University, Nanjing 211189, China; 230208352@seu.edu.cn; 2 Jiangsu Province Collaborative Innovation Center of Modern Urban Traffic Technologies, Southeast University, Nanjing 211189, China; 3 School of Transportation, Southeast University, Nanjing 211189, China

**Keywords:** connected and automated vehicles, CAV penetration, signalized intersection, capacity assessment, traffic state prediction

## Abstract

With the advancement of sensing, communication, and cooperative capabilities of connected automated vehicles (CAVs), the capacity and operational state of signalized intersections have become increasingly observable and suitable for prospective assessment. However, existing capacity models based on homogeneous traffic assumptions are insufficient to describe the capacity evolution of mixed traffic under varying CAV penetration levels. Motivated by this limitation, this study proposes a quantitative capacity estimation method for signalized intersections considering CAV penetration, serving as an evaluation and prediction baseline for intersection operations. The proposed method improves the CAV gain parameter and accounts for multiple typical car-following states in mixed traffic to derive equivalent headways and spacing coefficients, enabling a continuous estimation of intersection capacity with respect to CAV penetration. Using data from an actual signalized intersection, capacity and saturation trends are analyzed across different movement directions and traffic demand conditions. The results indicate a nonlinear increasing pattern of intersection capacity as CAV penetration rises, with distinct growth rates between straight-through and left-turn movements. The proposed approach provides an engineering-oriented reference for capacity estimation and traffic state prediction under mixed traffic conditions.

## 1. Introduction

### 1.1. Research Background

Recent advances in automated driving technologies, vehicle-to-everything (V2X) communication, high-performance computing, and intelligent sensing have profoundly reshaped the operation and management of transportation systems. As an integrated manifestation of these technologies, connected automated vehicles (CAVs) leverage onboard perception, autonomous decision-making, and vehicle-infrastructure cooperation to continuously sense and interact with their surrounding traffic environment. The technical scope and functional boundaries of CAVs have been systematically defined by the SAE (Society of Automotive Engineers) on-road automated driving levels [1]. At present, Level 2 driving assistance systems have been widely deployed, while Level 3 functions are gradually transitioning toward real-world applications.

In urban road networks, signalized intersections represent critical nodes characterized by concentrated traffic conflicts and complex operational mechanisms. Intersection efficiency plays a decisive role in overall network performance and is strongly influenced by traffic demand fluctuations, signal timing constraints, and vehicle interactions. Intersection capacity is a fundamental performance indicator that reflects traffic efficiency and congestion levels and supports traffic operation evaluation and management decisions [2]. Importantly, intersection capacity is not a static attribute but emerges from the combined effects of traffic composition, signal control strategies, and vehicle operational characteristics.

With the development of cooperative vehicle-infrastructure technologies, connected vehicle and infrastructure systems (CVIS) are establishing integrated information exchange networks among vehicles, road infrastructure, and control systems. Under CVIS environments, vehicles function not only as traffic participants but also as mobile sensing units capable of continuously reporting position, speed, vehicle type, and operational status. This mechanism substantially enhances the observability of intersection traffic states, enabling vehicle-type composition and car-following relationships to be explicitly identified and quantified. Such enhanced observability provides a technical foundation for moving beyond averaged assumptions toward more structured capacity characterization.

Considering fleet renewal cycles and market penetration trends, CAVs and human-driven vehicles (HVs) are expected to coexist for an extended period, forming mixed traffic conditions [3]. In this study, the term “heterogeneous traffic flow” is used to describe the traffic flow composition and car-following mechanisms involving both CAVs and HVs, while “mixed traffic” refers to the practical operating environment in which different vehicle types coexist. Significant differences in perception capability, reaction characteristics, and car-following behavior between CAVs and HVs are commonly characterized using the market penetration rate (MPR) [4]. As MPR increases, traffic operational characteristics and discharge processes at signalized intersections undergo structural changes, imposing new requirements on conventional capacity analysis approaches.

Existing studies on intersection capacity under mixed traffic conditions primarily rely on macroscopic empirical assumptions or simulation-based evaluations. These approaches remain limited in analytically characterizing traffic evolution under signal control constraints across different MPR levels [5,6]. In particular, within CVIS environments where vehicle types and car-following states are directly observable, traditional capacity estimation methods based on averaged headways or equivalent vehicle assumptions are unable to fully exploit the available observability information, thereby limiting analytical accuracy and interpretability. In addition to capacity-oriented studies, recent research on autonomous driving has increasingly emphasized robustness and safety under uncertain and disturbed environments, such as collision-free control and adaptive cruise control in the presence of external disturbances [7,8]. These studies highlight the importance of understanding traffic system performance under imperfect information and heterogeneous driving behaviors. From a macroscopic perspective, capacity estimation at signalized intersections under mixed traffic conditions provides a fundamental basis for evaluating the potential operational benefits and limitations of connected and automated vehicle deployment in such complex environments.

Against this background, this study investigates the capacity characteristics of heterogeneous traffic flow at signalized intersections under varying CAV penetration levels, leveraging the enhanced observability enabled by CVIS. By incorporating identifiable vehicle types and car-following states, the conventional capacity estimation framework is structurally refined to construct a quantitative capacity analysis method as a function of MPR. The proposed approach provides a methodological basis for intersection performance evaluation and supports subsequent research on cooperative signal control and optimization.

### 1.2. Related Works

Road capacity is a key indicator in urban traffic management, directly reflecting traffic efficiency, congestion levels, and the performance of the entire transportation system [9]. It serves as a basis for monitoring and evaluating traffic mobility [2]. Quantifying road capacity provides a foundation for alleviating traffic congestion, improving road utilization, and promoting the development of sustainable transportation systems [10].

Conventional intersection capacity models are primarily developed based on the operational characteristics of HV traffic flow and are therefore insufficient to capture the nonlinear changes introduced by CAVs. Existing studies have shown that CAVs have the potential to enhance traffic efficiency by reducing headways [11,12]; however, such improvements are influenced by factors including CAV penetration level and vehicle ordering within queues, leading to pronounced nonlinear and stage-dependent effects. Further research indicates that the introduction of CAVs affects not only individual vehicle behavior at the microscopic level but also the overall queue structure, phase utilization efficiency, and conflict-point dynamics at signalized intersections [13]. Under conventional traffic conditions, intersection efficiency is largely determined by lane configuration, turning lane allocation, and signal timing parameters such as green duration, phase combinations, and cycle length [14]. As CAVs are gradually integrated into traffic systems, their ability to maintain shorter headways, achieve smoother acceleration–deceleration profiles, and cooperate with signal control is theoretically expected to increase the number of vehicles discharged per unit time [5].

Currently, existing literature has analyzed the traffic capacity of CAV-based traffic flows from multiple perspectives [15,16]. However, the differences in behavior, dynamic characteristics, and interaction modes among different types of vehicles within heterogeneous traffic flows make their impact on road capacity more complex [17,18,19]. Such as, variations in reaction times, the statistical distribution of different driving behaviors, and the spatial dynamic changes between vehicles all contribute to this complexity [20,21,22].

Current methods for calculating the capacity of heterogeneous traffic flows are primarily derived from lane capacity and vehicle queuing theories. These methods consider the impact of different vehicle types on traffic flow density, speed, and capacity [13,23,24]. For instance, Zhou et al. [25] utilized a Gaussian Mixture Model (GMM) to derive the Fundamental Diagram (FD) of mixed HV and CAV traffic flows, while also revealing the effects of CAV penetration rates and platoon distributions on capacity. Although existing studies have explored the theoretical analysis of heterogeneous traffic flow capacity [26], most research often focuses on specific scenarios. These include analyses based on CAV platoons [27,28], such as investigating the impact of platoon size on capacity in heterogeneous traffic flows [29,30,31], as well as comparative studies on the effects of various dedicated lane configurations on the capacity of heterogeneous traffic flows [32,33].

Although these studies have laid a foundation for understanding the impact of heterogeneous traffic flows, there remains significant room for expansion in considering multiple vehicle types and broader traffic scenarios. In particular, signalized intersections, as highly challenging scenarios and critical nodes in urban road networks, present considerable complexity and importance in analyzing the capacity of heterogeneous traffic flows [34]. Specifically, aspects such as the regulation of signal phasing [35], the optimization of dedicated CAV lane configurations [5], and the potential impact of platoon formations on overall traffic efficiency in mixed traffic environments [36] require further in-depth investigation and analysis.

### 1.3. Research Content

Building on the preceding discussion of the limitations of conventional capacity analysis under mixed traffic conditions, this study focuses on signalized intersections as representative traffic scenarios characterized by high conflict intensity and strong operational constraints. Considering the long term coexistence of HVs and CAVs in intelligent transportation environments, this study establishes a quantitative framework for evaluating intersection capacity under mixed traffic conditions.

Unlike traditional approaches that rely primarily on macroscopic empirical assumptions or simulation based evaluation, the proposed framework is grounded in the enhanced traffic state observability provided by CVIS. Observable information on vehicle types, traffic composition, and signal control settings is explicitly incorporated to support an information enhanced characterization of the discharge process of mixed traffic under signal control constraints, with an emphasis on assessment rather than control prescription.

Within this framework, key control related factors, including CAV penetration level, signal cycle length, and phase configuration, are jointly considered to form a unified capacity analysis approach applicable to signalized intersections under mixed traffic conditions. By exploiting traffic state observability in CVIS environments, the proposed method enables the characterization of capacity evolution patterns and macroscopic constraint boundaries under different market penetration rates, providing an engineering interpretable reference for intersection performance assessment and operational potential analysis.

Based on the above framework, the main research content of this study can be summarized as follows:

- A quantitative capacity estimation framework is constructed to describe the evolution of signalized intersection capacity as a continuous function of CAV penetration under mixed traffic conditions.
- An information enhanced modeling approach is developed to incorporate identifiable vehicle types and typical car following states into capacity estimation, enabling a more refined representation of mixed traffic discharge behavior.
- The effects of movement type and signal phase configuration on capacity evolution are examined, with particular attention to the distinct responses of straight through and left turn movements to increasing CAV penetration.
- The proposed capacity estimation method is applied to an actual urban signalized intersection using observed traffic flow data and signal phase settings to evaluate its practical interpretability and applicability for intersection performance assessment.

The rest of the paper is as follows. Section 2 proposes a quantitative capacity estimation method for mixed traffic at signalized intersections. Section 3 applies the proposed method to a real-world intersection using officially collected traffic data. And the conclusions and future work are shown in Section 4.

## 2. Materials and Methods

### 2.1. CVIS Enabled Signalized Intersection Scenario

To support the observability-driven analysis and quantitative estimation of intersection capacity under mixed traffic conditions, a signalized intersection scenario based on CVIS is constructed in this study. Rather than serving as a platform for traffic control or optimization, the proposed scenario provides an information-rich environment for capturing traffic state characteristics and evaluating capacity evolution under varying levels of CAV penetration.

**Figure 1:**
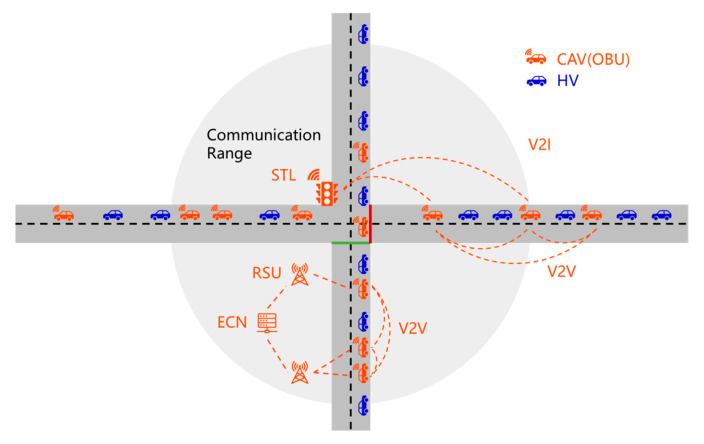
Signalized intersection scenario under the CVIS environment.

As illustrated in Figure 1, which is adapted from the authors’ previous work [37], the CVIS architecture consists of onboard units (OBUs) installed on CAVs, roadside units (RSUs), smart traffic lights (STLs), edge computing nodes (ECNs), and a cloud computing platform. Communication between CAVs and infrastructure is enabled through OBUs and RSUs, with RSUs forming an effective interaction domain around the intersection. This domain supports the acquisition of vehicle operating states, traffic composition, and signal control information. Vehicles entering and leaving the RSU coverage area define the spatial boundaries for traffic state observation within the intersection analysis.

Within this architecture, RSUs receive signal phase and timing information from STLs and disseminate it to vehicles, while ECNs integrate multi-source data collected from vehicles and infrastructure for local processing and coordination with cloud-based systems. Vehicle-to-vehicle (V2V) and vehicle-to-infrastructure (V2I) communication mechanisms enable continuous information exchange among traffic participants and roadside devices. In areas with sparse RSU deployment, cooperation between RSUs and nearby ECNs helps maintain the continuity of traffic information flows.

From the automation perspective, CVIS provides essential environmental perception and signal guidance information for CAVs, supporting more stable decision-making at signalized intersections. For higher automation levels, deeper vehicle–infrastructure cooperation can be supported, facilitating efficient autonomous operation in complex traffic environments.

### 2.2. Characteristics of Mixed Traffic at Signalized Intersections

In the signalized intersection scenario, vehicle operating behavior is jointly influenced by signal phases, queue formation and dissipation, and inter-vehicle interactions, resulting in discharge processes that exhibit pronounced periodic and stage-dependent characteristics [38]. In intelligent transportation environments, conventional HVs and CAVs differ substantially in perception mechanisms, decision-making processes, and longitudinal control precision. These differences lead the heterogeneous traffic flow to exhibit operational characteristics at signalized intersections that are distinct from those observed in mixed traffic conditions [39].

The Intelligent Driver Model (IDM) proposed by Treiber et al. [40] is a car-following model that incorporates both an acceleration tendency under free-flow conditions and a deceleration tendency associated with collision avoidance with the preceding vehicle. As formulated in Equation (1) and (2), the IDM effectively captures the car-following behavior of HVs and represents traffic states ranging from free flow to congested conditions.

(1) $$a_{n}\left(t\right)=a_{m}\left[1-{\left(\frac{v_{n}\left(t\right)}{v_{0}}\right)}^{\delta }-{\left(\frac{s^{*}\left(v_{n}\left(t\right),\Delta v_{n}\left(t\right)\right)}{\Delta x_{n}\left(t\right)}\right)}^{2}\right]f$$

(2) $$s^{*}\left[v_{n}\left(t\right),\Delta v_{n}\left(t\right)\right]=s_{0}+s_{1}\sqrt{\frac{v_{n}\left(t\right)}{v_{0}}}+T_{s}\cdot v_{n}\left(t\right)+\frac{v_{n}\left(t\right)\cdot \Delta v_{n}\left(t\right)}{2\sqrt{a_{m}\cdot b_{n}}}$$

where $a_{n}\left(t\right)$ denotes the acceleration under free-flow conditions (m/s^2^), $a_{m}$ is the maximum acceleration (m/s^2^), $v_{n}\left(t\right)$ represents the vehicle speed (m/s), $v_{0}$ is the desired speed (m/s), δ is the acceleration exponent, $s_{0}$ denotes the minimum static safety distance (m), $s_{1}$ is the speed-dependent safety distance (m), $s^{*}$ represents the desired minimum safety spacing (m), $\Delta v_{n}\left(t\right)$ is the relative speed with respect to the preceding vehicle (m/s), $\Delta x_{n}\left(t\right)$ denotes the actual inter-vehicle spacing (m), $T_{s}$ is the desired time headway (s), and $b_{n}$ represents the desired deceleration (m/s^2^).

To characterize the cooperative car-following behavior of CAVs under V2V communication conditions, this study adopts the widely used Cooperative Adaptive Cruise Control (CACC) model to describe the longitudinal control characteristics of CAVs [41]. In this model, the longitudinal acceleration of a vehicle is determined by its current speed, the actual spacing to the preceding vehicle, and the desired spacing. By incorporating motion information from the preceding vehicle, cooperative responses are achieved. The corresponding control laws are expressed in Equation (3) and (4).

(3) $$e=x_{i-1}-x_{i}-T_{\min}v_{i}$$

(4) $$v_{i}=v_{p}+k_{p}e+k_{d}\dot{e}$$

where e denotes the deviation between the actual spacing and the desired spacing (m); $x_{i-1}$ and $x_{i}$ represent the positions of the preceding vehicle and the following vehicle, respectively (m); $T_{\min}$ is the minimum safe spacing (m); $v_{i}$ denotes the speed of the following vehicle (m/s); $v_{p}$ is the vehicle speed at the previous time step (m/s); and $k_{p}$ and $k_{d}$ are the feedback gain parameters associated with spacing error (1/s^2^) and speed error (1/s), respectively. These gain parameters are calibrated based on field experiments conducted by the PATH program at the University of California, Berkeley, and are set to $k_{p}=0.45$ and $k_{d}=0.25$ [41].

During the saturated discharge phase at signalized intersections, although the instantaneous behavior of individual vehicles remains stochastic, the discharge process can be regarded as statistically stable when observed over multiple signal cycles. Its macroscopic characteristics can therefore be represented using aggregate indicators such as average headway. Consequently, intersection capacity analysis does not rely on fine-grained vehicle-level control models, but instead employs equivalent modeling approaches that characterize the average discharge behavior of different vehicle types.

Based on the above considerations, subsequent sections of this study adopt headway as the core indicator for characterizing the discharge behavior of heterogeneous traffic flow. By incorporating CAV penetration into the formulation, an equivalent headway model is constructed, which forms the basis for the proposed capacity estimation method for signalized intersections under mixed traffic conditions.

### 2.3. Quantitative Capacity Estimation Model for Signalized Intersections

Building on the preceding analysis of the nonlinear effects of CAV penetration and the multiple influencing factors inherent in signalized intersections, this chapter develops a quantitative capacity analysis method for signalized intersections under mixed traffic conditions. The proposed method is formulated with reference to the mixed traffic capacity modeling framework introduced by Chen et al. [26], which represents a widely cited and representative analytical approach in this research area. Since the present study builds upon this framework, it is adopted as the reference method for comparison, enabling a focused evaluation of the proposed improvements under consistent modeling assumptions.

During saturated discharge conditions, although the instantaneous car-following order and vehicle-level behaviors remain stochastic, the proportion of different vehicle types in mixed traffic can be regarded as stable when observed over multiple signal cycles. At this macroscopic level, the discharge characteristics of mixed traffic can be represented using average headway, allowing the contributions of HVs and CAVs to be approximated according to their respective penetration proportions.

Based on this statistical perspective, to incorporate car-following differences between HVs and CAVs into the estimation of saturation flow rates under mixed traffic conditions, this study adopts the concept of a weighted equivalent time headway to represent the average discharge interval at a given level of CAV penetration. Under the same movement type and lane group conditions, the equivalent headway of mixed traffic is expressed as a linear combination of representative headways for HVs and CAVs, weighted by their penetration proportions, as shown in Equation (3).

(5) $${\bar{h}}_{m}\left(\alpha \right)=\alpha \cdot h_{CAV,m}+\left(1-\alpha \right)\cdot h_{HV,m}$$

where $h_{CAV,m}$ and $h_{HV,m}$ denote the representative time headways (s) of CAVs and HVs, respectively; α represents the penetration rate of CAVs in the traffic flow. Equation (5) adopts a linear mixing approximation to represent the average headway under heterogeneous traffic conditions, with HV and CAV contributions weighted by MPR. This formulation serves as an analytical approximation to capture the expected average discharge behavior at the aggregate level, rather than explicitly modeling vehicle ordering or platoon formation in saturated queues. The robustness of this assumption is examined through the sensitivity analyses presented in Section 3.2.1.

**Figure 2:**
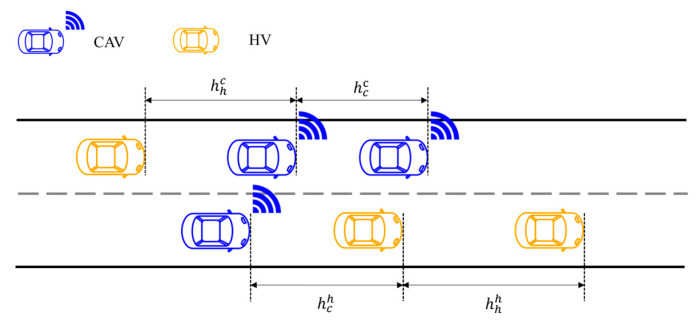
Headways in heterogeneous traffic flow.

To reflect the effects of vehicle type and turning behavior on discharge efficiency under mixed traffic conditions, this study adopts a scenario-based headway setting to characterize the car-following behavior of different vehicle types at signalized intersections. As discussed earlier, owing to higher longitudinal control precision and operational stability, CAVs are typically capable of operating with shorter acceptable headways than HVs. In addition, left-turn vehicles encounter more complex conflict environments and decision processes during discharge, resulting in larger average discharge intervals than those of straight-through movements. Accordingly, heterogeneous traffic flow headways vary across different movement scenarios, as illustrated in Figure 2 (adapted from the authors’ previous work [42]).

Based on the equivalent headway defined above, the saturation flow rate of mixed traffic can be expressed as the reciprocal of the headway ${\bar{h}}_{m}\left(\alpha \right)$ in Equation (6).

(6) $$s=\frac{3600}{{\bar{h}}_{m}\left(\alpha \right)}$$

To ensure engineering interpretability and methodological consistency, this study follows the capacity estimation framework proposed in the Highway Capacity Manual (HCM) [43] to quantify the discharge capacity of different lane groups at signalized intersections. As a widely recognized technical reference in traffic engineering, the HCM systematically summarizes the effects of signal timing, saturation discharge characteristics, and their impacts on intersection operational efficiency, and its methods have been extensively applied in intersection design and performance analysis.

According to the HCM definition, the capacity of a given lane group at a signalized intersection can be expressed as the product of the saturation flow rate and the proportion of effective green time within a signal cycle. Due to unavoidable time losses associated with vehicle start-up at the beginning of green phases and phase transitions, the effective green time is generally shorter than the nominal green duration. In addition, left-turn movements involve larger trajectory curvature and higher sensitivity to conflict conditions, which leads drivers to adopt more cautious acceleration behavior during start-up. To better reflect real-world traffic conditions, a start-up lost time term for left-turn movements is incorporated into the capacity formulation. The resulting intersection capacity is expressed as Equation (7):

(7) $$C_{s}=s\cdot \frac{g-\left(t_{L}+l\right)}{C}$$

where $C_{s}$ denotes the lane group capacity (veh/h/lane); s represents the saturation flow rate (veh/h/lane), reflecting the maximum discharge rate at which vehicles pass the stop line with a stable headway during the green phase; g is the green phase duration corresponding to the lane group (s); $t_{L}$ denotes the lost time (s), which mainly includes vehicle start-up loss and efficiency losses during phase transitions, and is set to 3 s; l represents the additional start-up lost time for left-turn movements, which is set to 1 s; and C denotes the signal cycle length (s).

Based on the above definitions, this study further develops intersection capacity calculation formulations applicable to mixed traffic conditions, as expressed in Equation (8) and (9).

(8) $$C_{h}=f\left(\alpha ,\varepsilon \right)=\frac{C_{s}}{1-\alpha \varepsilon }$$

(9) $$\varepsilon =1-\gamma -\left[\left(\beta^{C}-\gamma \right)+\left(\beta^{H}-\beta^{C}\right)\right]\cdot \left(1-\alpha \right)$$

where ε denotes the average critical spacing gain per CAV (referred to as the CAV gain), which represents the average proportion of spacing savings achieved by each CAV at critical headway conditions and reflects its marginal contribution to capacity improvement. γ is the spacing coefficient for CAVs following CAV leaders in mixed traffic, defined as the ratio of the corresponding headway to the critical spacing $s_{0}$ of HVs following HVs. $\beta^{C}$ denotes the spacing coefficient for CAVs following HV leaders, and $\beta^{H}$ represents the spacing coefficient for HVs following CAV leaders.

Based on the concepts of CAV gain and spacing coefficients adopted in the reference literature, the proposed capacity formulation restructures the original expression to improve its applicability under mixed traffic conditions. Specifically, the computation of the CAV gain ε is refined by replacing the original piecewise formulation, which only distinguishes between mixed traffic and fully automated traffic, with a continuous function explicitly related to the CAV penetration rate. This modification enables the CAV gain to more accurately characterize the nonlinear influence of MPR on intersection capacity.

**Figure 3:**
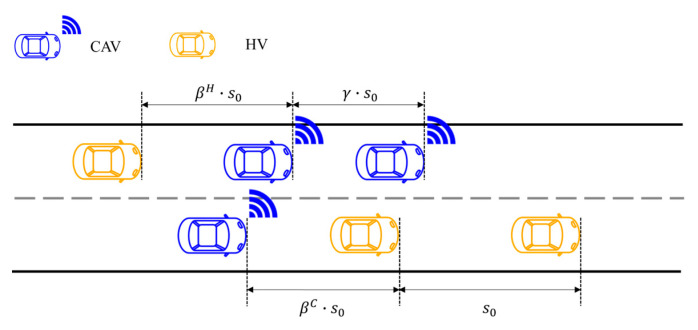
Headways and spacing coefficients in heterogeneous traffic flow.

Figure 3 (adapted from the authors’ previous work [42]) illustrates the spacing coefficient structure for mixed traffic, in which the headway definition in Figure 2 is decomposed by defining the headway $s_{0}$ of HVs following HVs as the baseline headway (m). The ratios of headways under the three mixed following scenarios, CAV following CAV, CAV following HV, and HV following CAV, to the baseline headway $s_{0}$ are denoted as γ, $\beta^{C}$, and $\beta^{H}$, respectively.

## 3. Results

To further demonstrate the applicability of the proposed capacity analysis method under real traffic conditions, a real urban signalized intersection is selected as the study object, and a case study is conducted based on observed traffic flow data and signal phase configurations. Leveraging the operational data available under CVIS conditions, this study quantitatively analyzes the variation patterns of intersection capacity under different CAV penetration scenarios and examines the corresponding changes in operational states. By applying the proposed method to a real-world intersection, this case study aims to illustrate its feasibility and interpretability for evaluating the operational potential of mixed traffic under realistic conditions, thereby providing a reference for subsequent intersection performance assessment and traffic management decision making.

### 3.1. Case Scenario and Data

#### 3.1.1. Case Scenario

**Figure 4:**
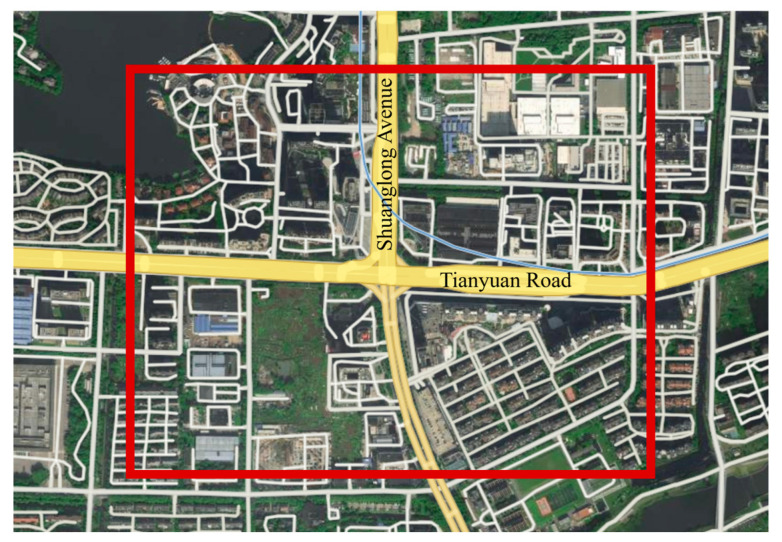
Selected study area of the signalized intersection.

The selected study site is the intersection of Shuanglong Avenue and Tianyuan Middle Road in Jiangning District, Nanjing, China, with geographic coordinates of longitude 118.821479 and latitude 31.928159. The study area is illustrated in Figure 4. Located in the core area of Jiangning District, the intersection is surrounded by dense commercial complexes and diverse land use types, exhibiting pronounced mixed traffic characteristics. Shuanglong Avenue is a north south oriented urban arterial road that carries major commuting traffic between the Jiangning urban area and metro corridor zones, while Tianyuan Middle Road is an east west oriented secondary road connecting several nearby residential and commercial areas. Influenced by commuting tidal effects, traffic demand at this intersection increases significantly during morning and evening peak periods, with distinct operational differences observed between off peak and peak conditions. As such, the intersection represents a typical congested urban signalized intersection.

**Figure 5:**
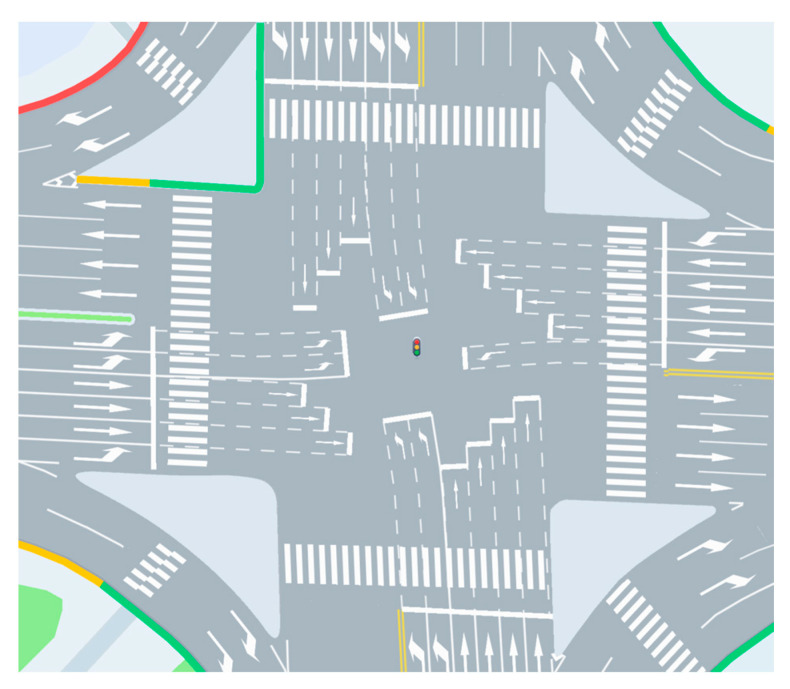
Lane configuration of the intersection.

Figure 5 illustrates the geometric layout and lane configuration of the studied signalized intersection, which defines the spatial scope of the capacity analysis. Right-turn movements from all approaches are diverted upstream and therefore excluded from signal control. As a result, only through and left-turn movements are considered in the subsequent capacity modeling and saturation analysis. During peak periods, the intersection experiences high traffic demand, characterized by reduced speeds and frequent queue formation, especially along the north–south arterial.

Overall, the studied intersection features a complex geometric layout, pronounced directional imbalance in traffic demand, and sustained congestion during peak periods. The combination of high arterial traffic volumes, multiple movement types, and strict signal control constraints imposes a substantial operational burden on the intersection. These characteristics make it a representative and meaningful case for examining capacity evolution and operational performance under mixed traffic conditions, particularly in the context of increasing CAV penetration.

#### 3.1.2. Case Data

Signal timing data for the intersection were provided by the Nanjing traffic management authority and include information on signal cycle length, green time, and yellow time for each phase. These data are used to construct the signal control logic in the simulation model. According to the observed timing plan, the intersection operates under a four phase fixed time control scheme. For each approach, straight through and left turn movements are released in separate phases, while right turn traffic is diverted upstream of the intersection and excluded from signal control.

**Figure 6:**
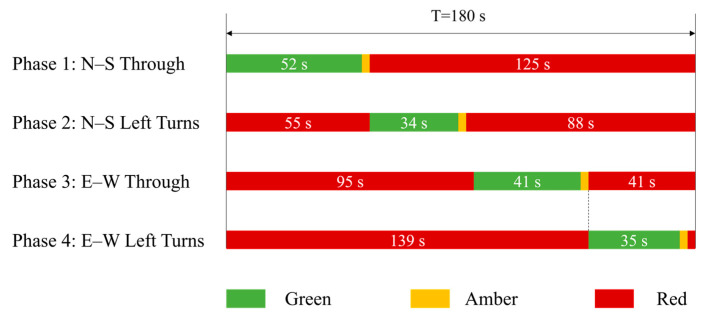
Signal phase sequence at the Shuanglong Avenue-Tianyuan Middle Road intersection.

As illustrated in Table 1, the four signal phases are arranged sequentially as north south straight through, north south left turn, east west straight through, and east west left turn movements. Figure 6 summarizes the duration of each phase. A 3 s all red interval is implemented after both the north south and east west movement phases, and the total signal cycle length is 180 s. This timing plan reflects the higher traffic demand and priority of the arterial road Shuanglong Avenue, with the green time allocated to north south straight through movements being significantly longer than that for east west movements, thereby ensuring sufficient capacity for the arterial direction.

The traffic flow data used in this study were obtained from real time operational monitoring records of the selected intersection for 24 October 2025, provided by the Nanjing traffic management authority. The data were automatically collected by license plate recognition camera systems installed at each approach of the intersection. Based on video detection and plate recognition technologies, the system is capable of accurately recording vehicle entry and exit times. These data capture the traffic operating characteristics of the intersection during a typical weekday and provide reliable empirical support for the simulation model.

To ensure the representativeness of the analysis, three typical time periods were selected, including the morning peak period from 8:00 to 9:00, the off peak period from 10:00 to 11:30, and the evening peak period from 18:00 to 19:00. Based on the passage records detected by the camera system, hourly traffic volumes for each approach were aggregated and summarized, as presented in Table 2. The results indicate that traffic demand in the north south direction along Shuanglong Avenue is significantly higher than that in the east west direction along Tianyuan Middle Road during peak periods, exhibiting a clear arterial dominated traffic pattern.

It should be noted that the case study data used in this paper are collected from an existing signalized intersection under current traffic conditions, where all vehicles are human-driven. Due to the lack of large-scale real-world mixed traffic data, different CAV penetration rates are introduced as hypothetical scenarios, and the proposed analytical formulation is applied to estimate intersection capacity under mixed traffic conditions. From this perspective, the analysis aims to provide a prospective assessment of capacity evolution with increasing CAV penetration, rather than merely reproducing observed mixed traffic states.

### 3.2. Analysis of Case Study Results

#### 3.2.1. Comparison of Method Performance

Movement direction is defined as $m\in \left\{TH,LT\right\}$, where *TH* represents through or right-turn movements and *LT* denotes left-turn movements. Representative headways for HVs and CAVs under different movement types are specified accordingly, and the corresponding parameter values summarized in Table 3. It should be noted that these headway values are not intended to be universal or fixed constants. Instead, they are selected as representative values within the headway ranges provided in [17], where headways are shown to vary across vehicle types and following states. For the purpose of facilitating comparative analysis and model implementation, specific headway values are adopted in this study to characterize different car-following scenarios under heterogeneous traffic conditions.

Similarly, based on the relevant descriptions in [26] and the characteristics of heterogeneous traffic flow, representative spacing values are adopted and summarized in Table 4. Based on the headway values under the four car-following states summarized in Table 4, the spacing coefficients are determined as γ=0.83, $\beta^{C}=0.87$, and $\beta^{H}=0.93$.

**Figure 7:**
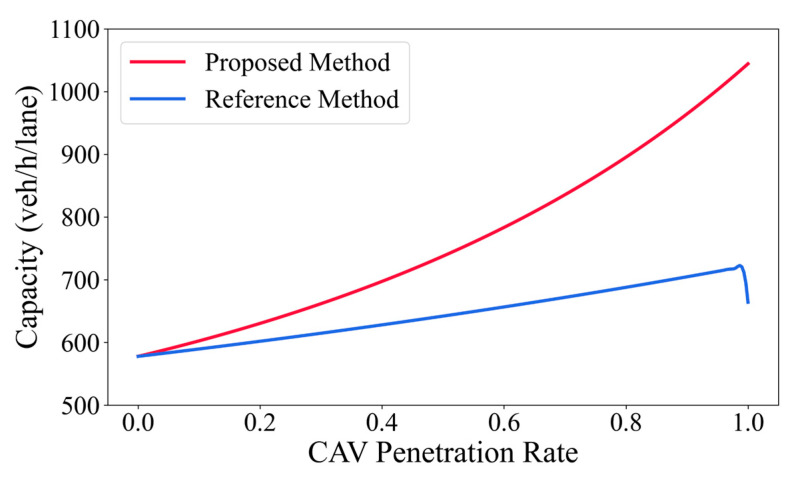
Capacity Variation with CAV Penetration.

This subsection focuses on the north–south straight through signal phase of the studied intersection (Figure 7) and compares the intersection capacity variations obtained using the reference method and the proposed improved method across different CAV penetration levels. It can be observed that the capacity curve produced by the reference method exhibits a pronounced non smooth pattern as penetration increases. Specifically, capacity increases during low to medium penetration levels but shows an abnormal sudden drop when penetration reaches 100%, decreasing from 720 veh/h/lane at MPR = 99% to 664 veh/h/lane. The abrupt capacity drop observed in the reference method at very high penetration levels can be attributed to the modeling treatment of the CAV gain parameter. In the reference formulation, the CAV gain, defined as the average critical spacing reduction introduced by CAVs, is specified using a piecewise expression and is assumed to be independent of the market penetration rate. As the penetration approaches unity, this assumption leads to a sudden adjustment of effective headway without a gradual transition, resulting in a non-smooth and physically inconsistent capacity estimate. By contrast, the proposed method explicitly accounts for the continuous influence of CAV penetration on capacity-related parameters, thereby avoiding such discontinuities near extreme penetration levels.

In contrast, the proposed improved method yields a stable and smooth growth trend in intersection capacity, increasing from 578 veh/h/lane to 1044 veh/h/lane. The improved method explicitly incorporates the observability and identifiability of vehicle operating states in mixed traffic under CVIS environments. In this setting, CAVs are able to accurately obtain information on surrounding vehicle types and operating states through vehicle to vehicle and vehicle to infrastructure communication, enabling the identification of different car following combinations in mixed traffic. Based on this capability, the improved method refines the specification of headways under four typical car following states and constructs an equivalent headway model directly linked to CAV penetration, which is then used to calculate saturation flow rates and intersection capacity.

Furthermore, the improved method revises the CAV gain within the capacity formulation process. Rather than relying on a single or coarse efficiency improvement assumption, the method differentiates among vehicle types and car following relationships to characterize the practical role of CAVs in enhancing queue dissipation efficiency, stabilizing discharge patterns, and reducing car following fluctuations. As a result, the resulting capacity curve exhibits a smooth and monotonic increasing trend with rising CAV penetration. This behavior is consistent with the evolution characteristics of mixed traffic at signalized intersections and reflects the continuous enhancement of overall system performance as the proportion of CAVs increases.

Notably, at medium to high penetration levels, the capacity improvement estimated by the proposed method is substantially greater than that obtained using the reference method. This difference arises from the more detailed consideration of headway variations across the four identifiable car following states under mixed traffic conditions, which allows the efficiency gains associated with increasing CAV penetration to be continuously and reasonably reflected at the capacity level.

To further examine the robustness of the proposed method with respect to representative headway settings, a sensitivity analysis is conducted by introducing two additional spacing scenarios around the baseline configuration. Instead of altering individual car-following relationships separately, the sensitivity design applies uniform absolute perturbations to all representative headway values to reflect potential variability in discharge behavior under different intersection and traffic conditions. Specifically, two supplementary scenarios are considered. In Case 1 (more compact spacing), all spacing values are reduced by 0.5 m relative to the baseline, representing a tighter discharge condition. In Case 2 (more conservative spacing), all spacing values are increased by 0.5 m, corresponding to a more cautious discharge condition. All other model parameters are kept unchanged across scenarios. The resulting spacing settings used in the sensitivity analysis are summarized in Table 5.

**Figure 8:**
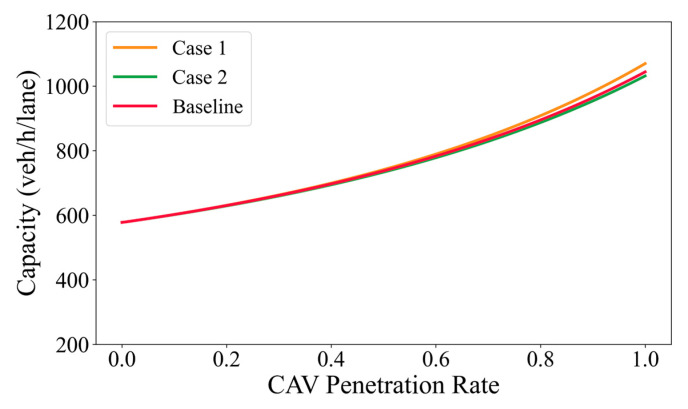
Capacity under different traffic speeds and penetration levels.

Figure 8 presents the sensitivity analysis results of intersection capacity under different representative headway settings. As shown, although slight variations in absolute capacity values can be observed among the three headway scenarios, the overall trends of capacity variation with respect to MPR remain highly consistent. In particular, the monotonic increase in capacity with increasing MPR is preserved across all scenarios, and the relative differences among the curves are limited over the entire MPR range. This indicates that the proposed capacity estimation method is not overly sensitive to the specific selection of representative headway values within a reasonable range. Therefore, the adopted headway settings can be considered applicable for the analyzed intersection conditions, and the main conclusions of this study remain robust against moderate variations in headway assumptions.

#### 3.2.2. Comparison of Capacity Across Different Movement Directions

**Figure 9:**
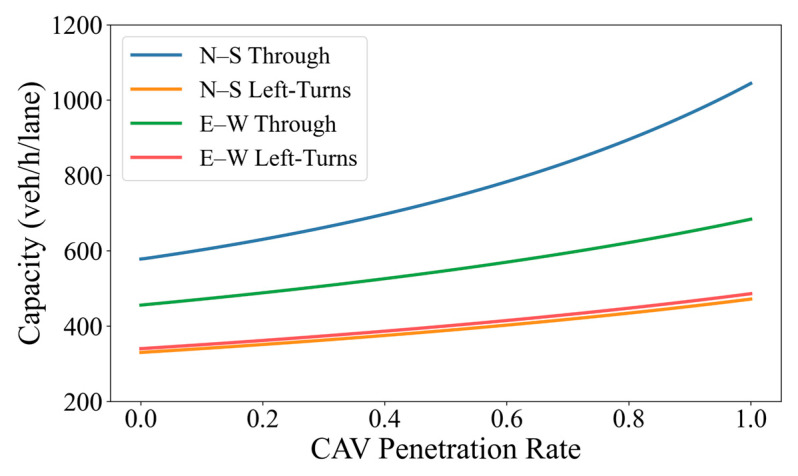
Capacity under different traffic speeds and penetration levels.

As described earlier, right turn traffic at the studied signalized intersection is diverted in advance. As a result, only four movement directions are retained within the intersection, including north to south (straight through), north to south (left turn), east to west (straight through), and east to west (left turn) movements, corresponding to four signal phases. The results from Figure 9 indicate that notable differences exist in capacity among the various movement directions. Among them, the north to south (straight through) movement exhibits the highest capacity, followed by the east to west (straight through) movement, while the north to south (left turn) and east to west (left turn) movements show comparatively lower capacity levels with similar magnitudes.

This result is consistent with the actual operational characteristics of the studied intersection. The north to south (straight through) movement corresponds to the primary arterial direction, carrying higher traffic demand and therefore being allocated a relatively longer green phase in practice, which supports a higher discharge capacity. By contrast, left-turn movements are typically associated with lower demand priorities, shorter effective green durations, and more complex conflict conditions at the intersection, leading to comparatively lower capacity levels.

To facilitate a direct comparison of how capacity varies with MPR across different movement directions, this study summarizes the capacity values at representative CAV penetration levels of 0%, 30%, 50%, 80%, and 100%, as reported in Table 6. The analysis shows that the capacities of all four movement directions increase monotonically with increasing CAV penetration rate, indicating that a higher proportion of CAVs contributes to overall improvements in intersection operational efficiency. However, the sensitivity to CAV penetration differs considerably among movement directions. The north to south (straight through) movement exhibits the most pronounced capacity improvement, with a relative increase of 80.6% from 0% to 100% MPR, which is substantially higher than that of the other three movements. The east to west (straight through) movement shows the second largest improvement, while the two left turn movements experience comparatively limited capacity gains.

This difference mainly arises from the varying extent to which different traffic flow can exploit the advantages of CAV. For straight through movements, vehicle operation is dominated by longitudinal car following behavior. Under such conditions, the advantages of CAVs in terms of reduced reaction time, smaller minimum safe headways, and improved speed stability can be fully realized, thereby substantially increasing the discharge rate per unit time. In contrast, left turn movements are constrained by signal control settings and conflict point distributions. Their operational efficiency is therefore more strongly governed by phase structure and intersection geometry, resulting in relatively weaker marginal improvements brought by CAV.

In addition, The results demonstrate that the relative ranking of capacities among different movement directions remains consistent across all penetration levels. This indicates that increasing CAV penetration does not alter the internal capacity structure of the intersection, but instead produces an overall upward shift in capacity for all movements. Under fixed signal timing and geometric conditions, the introduction of CAV thus primarily enhances efficiency rather than restructuring the capacity allocation among competing traffic streams.

Overall, the results indicate that increasing CAV penetration at the studied intersection leads to capacity improvements across all movement directions, with the most pronounced effects observed for the major straight through movements. This finding not only supports the validity of the adopted capacity estimation method under mixed traffic conditions, but also provides a quantitative basis for the subsequent implementation of differentiated control measures or priority strategies for different traffic flow directions.

#### 3.2.3. Saturation Analysis Under Different CAV Penetration Levels

As described earlier, right turn traffic at the studied signalized intersection is diverted in advance. As a result, only four movement directions are retained within the in

The degree of saturation represents the ratio of actual traffic flow to intersection capacity within a given time interval and is used to quantify the operational load level of a signalized intersection under specified control conditions. It is jointly determined by traffic demand and traffic supply. Traffic demand is typically characterized by observed arrival flow rates, reflecting the load imposed on the intersection by the external traffic system. Traffic supply is determined by the combined effects of saturation flow rate and effective green time, representing the maximum service capacity that the intersection can provide under the current signal control settings, as expressed in Equation (10).

(10) $$\mathrm{DoS}=\frac{v}{C_{h}}$$

where v denotes the traffic flow rate (veh/h/lane) and $C_{h}$ represents the capacity of mixed traffic (veh/h/lane).

**Figure 10:**
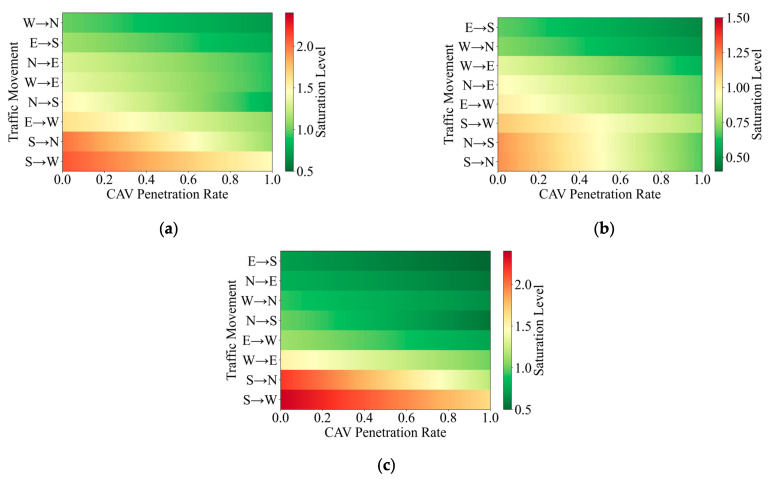
Degree of saturation under different traffic speeds and penetration levels. (**a**) Morning peak; (**b**) off peak; (**c**) evening peak.

Based on observed traffic flow data from the studied signalized intersection and the mixed traffic capacity estimation method proposed in this study, saturation levels for eight movement directions were calculated under different CAV penetration levels for three representative periods, including the morning peak, off peak, and evening peak. The results are presented in Figure 10.

From the overall comparison, it can be observed that under conditions without CAVs (MPR = 0%), several movement directions during the morning and evening peak periods exhibit pronounced high saturation or even oversaturation, with the evening peak being the most prominent. Specifically, during the evening peak period, the degree of saturation for the south to north (straight through) movement and the south to west (left turn) movement reaches 2.18 and 2.37, respectively, which are far above the saturation threshold of 1.0. This indicates a substantial mismatch between traffic demand and intersection capacity for the south approach during this period, making it a critical bottleneck that constrains overall intersection performance. In contrast, saturation levels across all movement directions during the off peak period remain relatively low, with only a few directions approaching saturation.

When examining trends associated with changes in CAV penetration, the degree of saturation for all movement directions across the three time periods consistently decreases as MPR increases. This indicates that a higher proportion of CAVs can effectively relieve intersection operational pressure under different traffic demand levels. However, the magnitude of this relief varies notably across time periods. The most significant improvements occur during the evening peak period, which can be attributed partly to the higher initial saturation levels and partly to the fact that under high demand conditions, the advantages of CAVs in reducing headways and enhancing discharge efficiency are more readily realized. Taking the south to north (straight through) movement as an example, its degree of saturation decreases from 2.18 (MPR = 0%) to 1.20 (MPR = 100%), corresponding to a reduction of 44.95%, which is the largest among all movement directions.

In comparison, the off peak period exhibits a more favorable operational state as CAV penetration increases. Based on Figure 10b, it can be inferred that when traffic flow consists entirely of CAVs, the degree of saturation for all movement directions during the off peak period can be maintained below 1.0. The maximum value occurs for the south to west left turn movement at 0.78, indicating that under moderate to low demand conditions, CAV has the potential to bring intersection operations into a stable and low congestion state.

To further examine the directional differences in how saturation varies with CAV penetration, the evening peak period is taken as an example. The degrees of saturation for all movement directions under different MPR conditions are compared, and the results are summarized in Table 7. It can be observed that substantial differences exist among movement directions during the evening peak period. Movements associated with the south approach exhibit the highest overall saturation levels, particularly the south to north (straight through) movement and the south to west (left turn) movement, both of which are markedly higher than those of other directions. This pattern is consistent with the practical characteristics of concentrated traffic demand at the south approach and the dominance of major outbound travel during the evening peak.

As CAV penetration increases, the degree of saturation for the south to west (left turn) movement decreases from 2.37 to 1.66, corresponding to a reduction of 29.96%. Although this represents a clear mitigation trend, the magnitude of the reduction is considerably smaller than that observed for the south to north (straight through) movement, which shows a reduction of 44.95%. The underlying reason lies not solely in differences in microscopic vehicle behavior, but more importantly in the degree of matching between traffic demand structure and capacity growth. The south to north (straight through) movement exhibits strong discharge continuity, with the largest proportion of green time at 28.89%. Under this condition, the increase in capacity resulting from higher CAV penetration can be effectively translated into serving existing peak demand, leading to a pronounced reduction in saturation. In contrast, the capacity growth of the south to west (left turn) movement is constrained by the limited proportion of left turn green time at 18.89% and by phase transition constraints. Even at higher levels of CAV penetration, the additional capacity remains insufficient to fully accommodate the concentrated demand, resulting in a relatively limited improvement in saturation. These findings indicate that under high demand conditions, the mitigation effects of increasing CAV penetration differ substantially across movement directions.

Overall, the analysis indicates that the proposed mixed traffic capacity calculation method provides a comparable reference baseline for evaluating the operational state of signalized intersections. The results show that intersection capacity is jointly influenced by multiple factors, including movement direction and signal phase configuration. Notable differences exist between straight through and left turn movements in terms of capacity levels and their response characteristics to changes in CAV penetration. In addition, green phase duration exhibits a clear positive effect on capacity improvement, with longer green times leading to larger capacity gains as MPR increases.

## 4. Conclusions

This study examines the operational performance of signalized intersections under mixed traffic conditions where CAVs coexist with HVs, with a focus on how intersection capacity and operational states evolve across different CAV penetration levels. To overcome the limitations of existing studies in providing continuous quantitative assessments of mixed traffic capacity, a CAV-penetration-based capacity estimation method is proposed. The method establishes a unified and comparable analytical baseline for evaluating intersection operations under mixed traffic conditions.

From a methodological perspective, the study builds upon conventional capacity calculation frameworks by refining the CAV gain parameter and distinguishing car following states under different vehicle combinations. This leads to a continuous capacity quantification approach as a function of CAV penetration. The method systematically characterizes the dynamic evolution of capacity under different car following relationships and signal phase conditions, and avoids the potential evaluation distortions that may arise from segmented empirical models at high penetration levels. As a result, it provides a more realistic representation of mixed traffic operations at signalized intersections.

Based on a real-world intersection case study, the results indicate that increasing CAV penetration leads to an overall increase in intersection capacity and a reduction in saturation levels across all movement directions, with varying magnitudes. Straight-through movements, benefiting from longer green phases and more continuous discharge processes, exhibit greater sensitivity to capacity growth with rising CAV penetration. In contrast, left-turn movements remain more constrained by phase structure and turning conflicts, resulting in comparatively limited capacity gains and saturation relief. These results confirm the validity of the proposed capacity estimation method under mixed traffic conditions. Future research may extend the proposed framework to different intersection geometries and demand patterns, while also incorporating stochastic queue compositions or platoon formation effects to further assess its applicability and potential nonlinear impacts under saturated mixed traffic conditions.

## Figures and Tables

**Table 1:** Signal timing scheme at the Shuanglong Avenue and Tianyuan Middle Road intersection.

Phase	Direction	Movement Type	Green Time (s)	Yellow Interval (s)	Red Time (s)
Phase 1	North-South	Through	52	3	88
Phase 2	North-South	Left Turn	34	3	88
Phase 3	East–West	Through	41	3	98
Phase 4	East–West	Left Turn	35	3	98

**Table 2:** Traffic volumes by movement direction during different time periods at the intersection.

Movement Direction	Time Periods		
	Morning Peak (8:00–9:00)	Off Peak (10:00–11:30)	Evening Peak (18:00–19:00)
South to north (Through)	4633	4226	5032
South to west (Left Turn)	1381	1106	1563
North to south (Through)	3468	4207	2331
North to east (Left Turn)	858	953	534
West to east (Through)	2493	2438	2784
West to north (Left Turn)	682	739	630
East to west (Through)	2974	2764	2045
East to south (Left Turn)	764	695	487

**Table 3:** Representative headways in heterogeneous traffic flow.

$h_{HV,TH}$	$h_{CAV,TH}$	$h_{HV,LT}$	$h_{CAV,LT}$
1.8	1.2	2.0	1.4

**Table 4:** Critical spacing in heterogeneous traffic flow.

HV-HV	HV-CAV	CAV-HV	CAV-CAV
7.5	7.0	6.5	6.2

HV–HV, HV–CAV, CAV–HV, and CAV–CAV denote four leader–follower configurations.

**Table 5:** Critical spacing in eterogeneous traffic flow under different scenario.

Scenario	HV-HV	HV-CAV	CAV-HV	CAV-CAV
Baseline	7.5	7	6.5	6.2
Case 1	7	6.5	6	5.7
Case 2	8	7.5	7	6.7

**Table 6:** Capacity of mixed traffic at the signalized intersection (veh/h/lane).

Movement Direction	CAV Penetration Rate				
	0%	30%	50%	80%	100%
North–South (Through)	578	662	738	895	1044
North to east (Left Turn)	330	363	389	435	472
East to west (Through)	456	507	547	622	684
East to west (Left Turn)	340	374	400	448	486

**Table 7:** Degree of saturation for each movement direction during the evening peak.

Movement Direction	CAV Penetration Rate				
	0%	30%	50%	80%	100%
South to north (Through)	2.18	1.90	1.71	1.41	1.20
South to west (Left Turn)	2.37	2.15	2.01	1.80	1.66
North to south (Through)	1.01	0.88	0.79	0.65	0.56
North to east (Left Turn)	0.81	0.74	0.69	0.61	0.57
West to east (Through)	1.53	1.37	1.27	1.12	1.02
West to east (Left Turn)	0.93	0.84	0.79	0.70	0.65
West to north (Through)	1.12	1.01	0.93	0.82	0.75
West to north (Left Turn)	0.72	0.65	0.61	0.54	0.50

## Data Availability

The data that support the findings of this study are available from the corresponding author upon reasonable request.

## Abbreviations

The following abbreviations are used in this manuscript:

V2X	Vehicle-to-everything
CAV	Connected and automated vehicle
SAE	Society of Automotive Engineers
CVIS	Connected vehicle and infrastructure system
HV	Human-driven vehicle
MPR	Market penetration rate
OBU	On-board unit
RSU	Road side unit
STL	Smart traffic light
ECN	Edge computing node
V2V	Vehicle-to-vehicle
V2I	Vehicle-to-infrastructure
IDM	Intelligent driver model
CACC	Cooperative adaptive cruise control
